# An accurate paired sample test for count data

**DOI:** 10.1093/bioinformatics/bts394

**Published:** 2012-09-03

**Authors:** Thang V. Pham, Connie R. Jimenez

**Affiliations:** OncoProteomics Laboratory, Department of Medical Oncology, VU University Medical Center De Boelelaan 1117, 1081 HV Amsterdam, The Netherlands

## Abstract

**Motivation:** Recent technology platforms in proteomics and genomics produce count data for quantitative analysis. Previous works on statistical significance analysis for count data have mainly focused on the independent sample setting, which does not cover the case where pairs of measurements are taken from individual patients before and after treatment. This experimental setting requires paired sample testing such as the paired *t*-test often used for continuous measurements. A state-of-the-art method uses a negative binomial distribution in a generalized linear model framework for paired sample testing. A paired sample design assumes that the relative change within each pair is constant across biological samples. This model can be used as an approximation to the true model in cases of heterogeneity of response in complex biological systems. We aim to specify the variation in response explicitly in combination with the inherent technical variation.

**Results:** We formulate the problem of paired sample test for count data in a framework of statistical combination of multiple contingency tables. In particular, we specify explicitly a random distribution for the effect with an inverted beta model. The technical variation can be modeled by either a standard Poisson distribution or an exponentiated Poisson distribution, depending on the reproducibility of the acquisition workflow. The new statistical test is evaluated on both proteomics and genomics datasets, showing a comparable performance to the state-of-the-art method in general, and in several cases where the two methods differ, the proposed test returns more reasonable *p*-values.

**Availability:** Available for download at http://www.oncoproteomics.nl/.

**Contact:** t.pham@vumc.nl

## 1 INTRODUCTION

Recent technology platforms in proteomics and genomics produce count data for quantitative analysis. In proteomics, the number of MS/MS events observed for a protein in the mass spectrometer has been shown to correlate strongly with the protein's abundance in a complex mixture (Liu *et al.*, 2004). In genomics, next-generation sequencing technologies use read count as a reliable measure of the abundance of the target transcript (Wang *et al.*, 2009). Statistical analysis of count data is therefore becoming increasingly important.

Previous works using a beta-binomial model (Pham *et al.*, 2010) or a negative binomial model (Anders and Huber, 2010; Robinson *et al.*, 2010) have focused on an independent sample setting. Nevertheless, consider a study where data are measured from each patient before and after treatment. The measurements are no longer independent. Another frequently used design is to compare the gene/protein expression levels between matched pairs of cancer and normal tissues. These experimental designs require paired sample testing such as the paired *t*-test used for continuous measurements.

Due to the lack of a proper statistical test, recent efforts in proteomics (van Houdt *et al.*, 2011) and genomics (Tuch *et al.*, 2010) have resorted to calculating fold changes within each sample and subsequently using a rule-based system to identify differential markers. The rules have to take into account such issues as difference in variation at regions of low and high abundance. While this approach might be applicable for a discovery study with a small sample size (e.g. *N* = 3 pairs), it possesses no concept of statistical significance for generalized inference. In this article, we aim to develop a statistical method for analysis of paired samples for count data.

For ease of presentation, we consider an experiment in which each sample pair consists of a pre-treatment measurement and a post-treatment measurement. One is interested in the relative change in abundance due to the treatment for each protein across biological samples. To this end, we derive from each protein in each sample pair a 2×2 contingency table containing a pre-treatment count and a post-treatment count as well as total sample counts for normalization. The treatment effect and statistical significance can be computed using the Fisher's exact test or the *G*-test for each contingency table. However, combining information from multiple tables to obtain a common effect and a confidence estimate is non-trivial, and has been a central problem in the field of meta-analysis.

This article proposes a new technique to estimate a common effect and significance analysis for multiple contingency tables. The method uses ratio of two Poisson distributions, leading to a binomial distribution parameterized by a single random effect variable. The random effect is subsequently modeled with an inverted beta distribution, resulting in an inverted beta binomial model for the observed count data. The new statistical test is therefore called the inverted beta binomial test.

The article is organized as follows. Section 2 presents mathematical notations, the concept of common treatment effect and previous approaches to the problem. Section 3 presents the new statistical test and its implementation. Experimental results are presented in Section 4. Section 5 concludes this article.

## 2 TREATMENT EFFECT IN PAIRED SAMPLE TEST

Let *a* and *b* denote the counts of a specific protein measured before and after treatment and *t_a_* and *t_b_* be the total count of all proteins measured, respectively. One can construct a 2×2 contingency table for this protein as in Table 1.

**Table 1.** A 2×2 contingency table for a single protein in each sample pair

	Before treatment	After treatment
Protein of interest	*a*	*b*
All other proteins	*c*	*d*
Total sample count	*t_a_ = a+c*	*t_b_ = b+d*

In paired sample testing, the observed treatment effect *f* is the fold change in abundance after normalization for total sample count
(1)
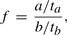

where *a/t_a_* and *b/t_b_* are observed fractions of the protein present in the sample before and after treatment, respectively. Let the true fold change be *φ*= *π_a_/π_b_*, where *π_a_* and *π_b_* are the true fractions of the protein. The observed values and true values are related by a generative model of the data. Assume that the count *a* and *b* are generated according to Poisson distributions with mean *π_a_t_a_* and *π_b_t_b_*, respectively
(2)
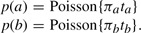

Let **x** = (*a,b,t_a_*,*t_b_*) be a vector of observed data. Consider a set of *N* sample pairs {**x***_n_*}. We are interested in estimating a common effect 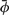 and a confidence of this estimation. When *N* = 1, one can apply the Fisher's exact test or the *G*-test for significance analysis. Combining multiple sample pairs (*N >* 1) is a non-trivial problem. It is a central problem in meta-analysis in statistics literature and still is an active research topic, especially in the context of combining results from multiple clinical trials, where the calculation of the so-called relative risk is identical to Equation (1).

The Mantel–Haenszel (MH) test (Mantel and Haenszel, 1959) and the DerSimonian–Laird test (DSL) (DerSimonian and Laird, 1986) are two classical methods to analyze combination of 2×2 contingency tables. Assuming that the effects are constant over biological samples, 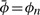 for *n* = 1,*...,N*, the MH method can be used for testing a null hypothesis of no treatment effect 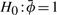. When applying the MH test to a spectral count dataset in proteomics (see Section 4.2), we obtained several unsatisfactory results. For instance, the MH test returns a very low *p*-value (*<*0.00001) for a protein with normalized counts (13, 26), (188,73) and (69, 75) for three sample pairs. (Normalized counts are obtained by scaling all samples to a common total count and rounding. They are used for ease of presentation and not for calculation of the test). Here, the effects are opposite for the first two sample pairs and approximately one in the third. Thus, the result is not intuitive.

**Fig. 1.**
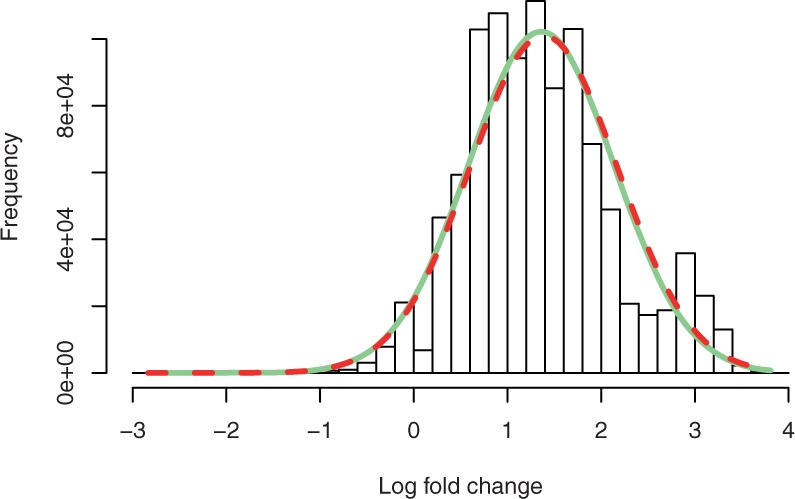
Example of an observed **x** = (2,8,30 000,30 000) with histogram of log fold changes derived from 1 million pairs of Poisson distribution with mean 2 and 8. A correction term of 0.5 was used. The normal approximation using observed fold change and (1/*a*+1/*b*+1/*c*+1/*d*)-variance (dashed line) follows the normal approximation of the simulated data (solid line). Both do not approximate well the multimodal histogram

The DSL method models log *f_n_* as realizations from normal distributions
(3)
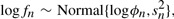

where *s_n_*^2^ are the sampling error. Furthermore, log *ϕ_n_* are drawn from a normal distribution Normal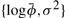 where *σ*^2^ is the variance across biological samples. As a result, log*f_n_* are drawn from a normal distribution Normal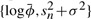. A frequently used estimation for *s_n_*^2^ is (1*/a_n_* + 1*/b_n_* + 1*/c_n_* + 1*/d_n_*). For small counts, it is known that the normal approximation in Equation (3) is problematic. In addition, a correcting term is required to compute the observed log *f_n_*. A recent work (Hamza *et al.*, 2008) has demonstrated that exact modeling of within study (in our setting, within patient or technical variation) is superior to normal approximation. Fig. 1 illustrates a model of variation for an observed **x** = (*a* = 2,*b* = 8,*t_a_* = 30 000,*t_b_* = 30 000). The histogram of fold changes derived from 1 million pairs is multimodal (raw count numbers were generated with *π_a_* = *a/t_a_* and *π_b_* = *b/t_b_* in a Poisson model and fold changes were calculated with a correction term of 0.5). The normal approximation using observed fold change and (1*/a*+1*/b*+1*/c*+1*/d*)-variance (dashed line) follows the normal approximation of the simulated data (solid line), but both do not approximate well the multimodal histogram.

A modern approach to paired sample testing for count data is represented by a recent extension of the edgeR method (McCarthy *et al.*, 2012). It uses a negative binomial distribution to model total variation. It is known that for a fixed dispersion, a negative binomial distribution can be placed in a generalized linear model framework. Thus, a paired test can be performed by providing an appropriate design matrix with patient as predictors, resulting in a fixed effect model for *ϕ*, that is 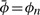, ∀*n*, as in MH.

In the following we propose a novel method consisting of a random effect model and exact modeling for technical variation. We will compare the new method with the extension of edgeR (McCarthy *et al.*, 2012) as it represents the current state-of-the-art in paired sample testing for count data.

## 3 THE INVERTED BETA BINOMIAL MODEL

According to Bross (1954), when the counts are Poisson distributed as in (2) and *a*+*b* is considered fixed, the ratio *ϕ*= *π_a_/π_b_* is a single parameter of a binomial distribution of the counts as follows:
(4)
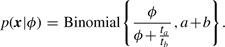


We model *ϕ* as a random variable generated from an inverted beta distribution as follows:
(5)
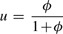

(6)
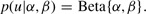


The beta distribution has support on (0,1) and is characterized by two parameters *α* and *β*. For *α >* 1 and *β >* 1, the distribution is unimodal with mean *α/*(*α* +*β*). Thus, for our application, a fold change *ϕ*= 1 is equivalent to *u* = 0.5, which coincides with the mean of a beta distribution with *α* = *β*.

**Fig. 2.**
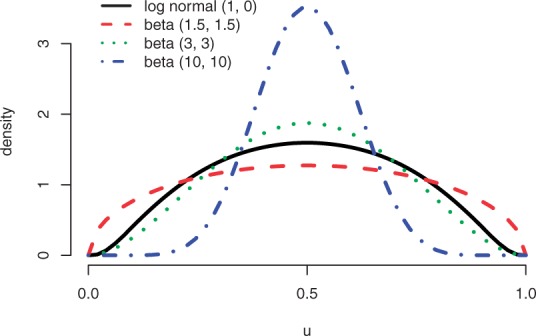
Comparison of the normal and the beta distribution for the random effect *ϕ*. The solid line represents a normal distribution of log*ϕ* with zero mean and unit variance when transformed to the *u*-space (see text). The variance of the beta distribution reduces as (*α* +*β*) increases. All distribution concentrates around *u* = 0.5, which is equivalent to *ϕ* = 1

Naturally, a normal distribution can be used to model the random effect *ϕ*, resulting in a normal-binomial model, analogous to the model used in Hamza *et al.* (2008). Figure 2 illustrates the similarity of the normal distribution and the beta distribution. The solid line depicts the distribution of *u* when log*ϕ* follows a normal distribution with zero mean and unit variance. All distributions concentrate around 0.5. A beta distribution with *α* = *β* = 1.5 exhibits a larger variance than that of the transformed normal distribution, and the variance of the beta distribution reduces as (*α* +*β*) increases.

Both normal-binomial model and inverted beta-binomial model belong to the class of hierarchical modeling for which maximum likelihood optimization is difficult because of the integrations involved. For one-dimensional optimization, however, numerical quadratures provide accurate approximation. For the normal-binomial model, Hamza *et al.* (2008) use a Gaussian quadrature as implemented in a commercial system SAS. This article focuses on the inverted beta distribution since the resulting inverted beta binomial distribution has a property that in case *t_a_* = *t_b_*, for example when the data have been normalized, the marginal distribution has a closed form known as the beta-binomial distribution (Skellam, 1948), and efficient software for maximum likelihood optimization is available (Pham *et al.*, 2010). Furthermore, for large fold changes, we find that in our implementation, the inverted beta binomial model using a Gauss–Jacobi quadrature (see below) is numerically more stable than the normal-binomial using a Gaussian quadrature.

From Equations (4)–(6), we obtain a marginal distribution
(7)
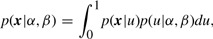

where
(8)
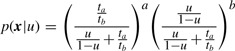

(9)
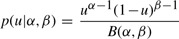

and *B*(*α,β*) is the beta function.

Let *z* = 2*u*−1. The marginal distribution of ***x*** in (7) can be approximated by a Gauss–Jacobi quadrature (Golub and Welsch, 1969) as follows:
(10)
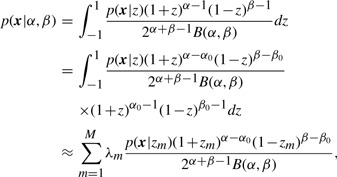

where (*λ_m_*,*z_m_*) are *M* points of a Gauss–Jacobi quadrature with parameters (*α*_0_ − 1,*β*_0_ − 1), *α*_0_ *>* 0,*β*_0_ *>* 0.

The log-likelihood can be expressed as a log-sum-exp function
(11)
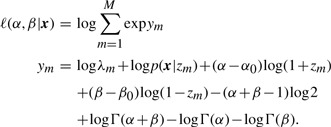


Given *N* sample pairs **x***_n_*, we can compute the maximum likelihood estimate 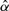 and 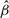

(12)
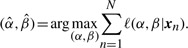

The optimization problem (12) can be efficiently solved since all partial derivatives of ℓ up to second order can be computed analytically.

The ratio *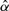 / 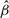
* provides an estimate for the common fold change 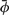. The likelihood-ratio test can be used for significance analysis. To test a null hypothesis *H*_0_ : *α* = *β* against the alternative hypothesis *H*_1_ : *α* ≠ *β*, we compute a statistic equal two times the difference of maximum log likelihoods estimated under the null hypothesis and the unconstrained model
(13)
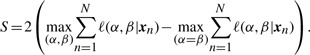

The distribution of the statistic under the null hypothesis is approximated by the *χ*^2^ distribution with one degree of freedom.

### 3.1 Adapting technical variation

**Fig. 3.**
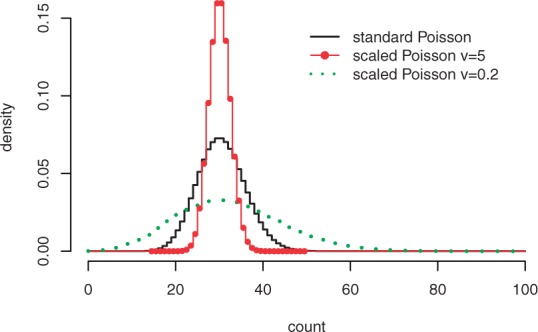
Three exponentiated Poisson distributions with modes at 30. The solid line (*v* = 1) represents the standard Poisson distribution. The distribution with a higher value of *v* (*v* = 5) is more tightly distributed around its mode. A lower value of *v* (*v* = 0.2) is suited for data exhibiting more variation

An advantage of separating technical variation from biological variation is that we can assess the underlying acquisition platform and subsequently adapt the statistical model accordingly. Informally, technical variation represents the distribution of the counts as if a single pair was to be measured multiple times. For count data, a Poisson distribution as in Equation (2) can be used, reflecting the observation that variation tends to increase for higher abundance proteins. Nevertheless, the relationship between variance and average abundance is settled for a Poisson distribution, the variance is equal to the mean. For a more flexible mean–variance relationship, we can use an exponentiated Poisson distribution
(14)
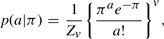

with *v >* 0 and *Z_v_* is a normalizing factor that depends on the value of *v* only. When *v* = 1 we have the standard Poisson distribution. The extra parameter *v* provides flexibility with regard to mean–variance relationship, while maintaining the mode of the distribution at ⌊*π*⌋. Figure 3 shows three distributions with *π* = 30 and *v* = 0.2,1,5. It can be seen that for more reproducible data, a higher value of *v* can be used, whereas for data exhibiting extra dispersion, a lower value of *v* is more appropriate.

It can be shown that the ratio of two exponentiated Poisson distributions with fixed *v* leads to an exponentiated Binomial distribution instead of Equation (4). The optimization procedure is identical to the standard Poisson model with an exception in evaluating the likelihood in Equation (11).

It should be stressed that deviating from the standard Poisson distribution requires careful experimental support, for example from independent technical runs. We will show the possibility of adapting technical variation in an example. For experiments on real-life datasets, we use the standard Poisson distribution.

## 4 RESULTS

### 4.1 A numerical example

Consider a gene from the RNA-Seq dataset in 4.2 (expression count/total sample count) in three sample pairs as follows:

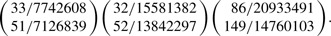

The average total count across all samples is 1 33 31 120. After being normalized to this average count and rounded, the expression counts of the three pairs are (57, 95), (27, 50) and (55, 135). Again, the normalized counts are used for a convenience of presentation only. The raw values are used for calculation in all exact tests examined in this article.

**Fig. 4.**
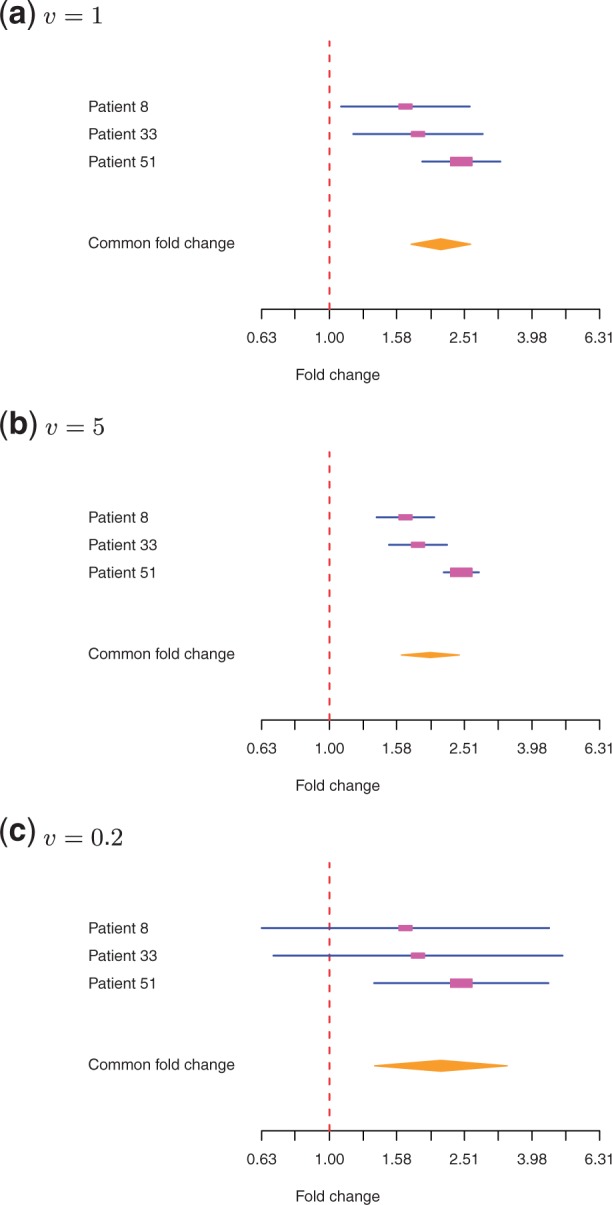
Combing evidence from three patients for a gene with rounded, normalized counts of (57, 95), (27, 50) and (55, 135). The forest plots show estimation of individual fold changes and confidence intervals for three models of technical variation: (**a**) a standard Poisson distribution, (**b**) an exponentiated Poisson distribution with *v* = 5, and (**c**) an exponentiated Poisson distribution with *v* = 0.2. The lines are confidence intervals. The rectangles indicate the strength of contribution of each sample to the common estimation. The diamond shape represents the common fold change and its confidence interval

The ibb test returns an estimated common fold change 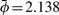, a test statistic *S* = 8.237, and a *p*-value = 0.004. Figure 4a shows a forest plot for the result. A forest plot is a dedicated visualization tool in meta-analysis, showing estimation of fold changes and confidence intervals for individual samples and group, (see Thomas Lumley. *rmeta: Meta-analysis*, 2009. R package version 2.16.). Interval estimation was based on the Hessian at the optimal solution of Equation (12).

Figure 4b and c shows the estimation when the technical variation is adapted using an exponentiated Poisson distribution with *v* = 5 and *v* = 0.2, respectively. It can be seen that the confidence intervals concentrate around the point estimation for a high technical reproducibility with *v* = 5. On a less reproducible platform with *v* = 0.2, the uncertainty is more pronounced with confidence intervals of two individual samples containing one (representing no effect). The common estimation with *v* = 0.2 gives a *p*-value = 0.016, higher than the standard Poisson model, but still significant at 5% cutoff.

### 4.2 Comparison with existing methods on real-life datasets

We compare the results of edgeR and ibb on two real-life datasets. The first dataset (van Houdt dataset) is a comparative proteomics analysis of an *in vitro* model of colon cancer stem cells and differentiated tumor cells (van Houdt *et al.*, 2011). Each sample pair was derived from freshly resected liver metastases. In total, more than 3000 proteins were identified and quantified by spectral counting. The average total count is about 27 000. On this dataset, common fold changes were estimated using the MH method. The significance analysis with MH was, however, not satisfactory as several proteins had highly significant *p*-values despite of having opposite regulations among the three sample pairs. Subsequently, differential proteins were determined using cutoffs on unidirectional fold changes of all the pairs.

The second dataset (Tuch dataset) is an RNA-Seq dataset in a study of the development of oral squamous cell carcinoma (OSCC) (Tuch *et al.*, 2010). Three OSCC tumors and three matched normal tissues were analyzed to obtain transcriptome read counts. The average total count is approximately 13×10^6^. Again, fold changes were used to identify differentially expressed genes. Recently, this dataset has been used to demonstrate the power of the extension of the edgeR method for a paired sample design (McCarthy *et al.*, 2012).

**Fig. 5.**
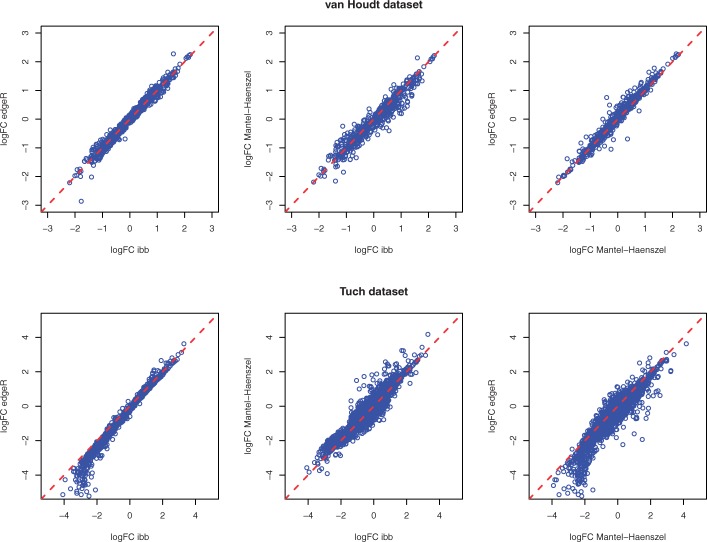
Fold changes estimated by inverted beta binomial, edgeR and MH on the van Houdt dataset and Tuch dataset

Figure 5 shows the fold changes of all genes/proteins not having black and white regulation (all zeros in one group) in the two datasets as estimated by ibb, edgeR and MH. It can be seen that fold change estimation is stable across all three methods. On the Tuch dataset, we observe that edgeR tends to produce a lower fold change than both ibb and MH for a number of down-regulated genes. We speculate that this is due to the gene-wise dispersion smoothing implemented in edgeR. For the van Houdt dataset where the average count for each protein is relatively small, the smoothing procedure has no clear effect.

**Fig. 6.**
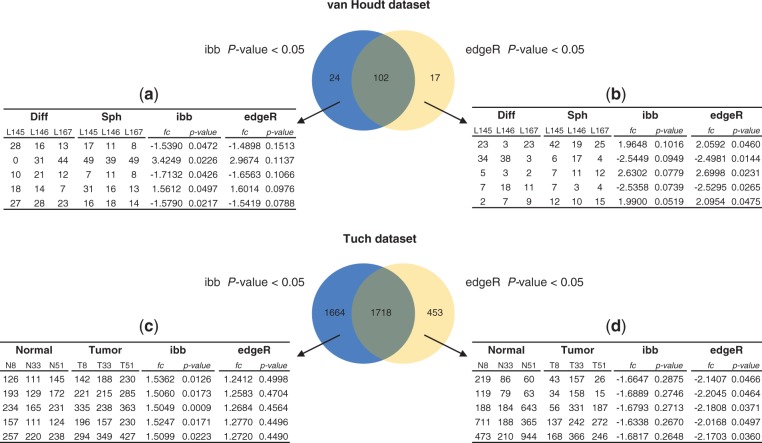
Overlap analysis of the proteins and genes with *p*-values < 0.05 by either ibb or edgeR. The counts in the tables are rounded normalized values. (**a**) Five proteins having highest edgeR *p*-value and ibb *p*-value < 0.05. (**b**) Five proteins having highest ibb *p*-value and edgeR *p*-value < 0.05. (**c**) Five proteins having highest edgeR *p*-value, ibb *p*-value < 0.05, and absolute fold change > 1.5. (**d**) Five proteins having highest ibb *p*-value, edgeR *p*-value < 0.05 and absolute fold change > 1.5

Next, we examine the counts of individual proteins/genes where the *p*-values returned by ibb and edgeR differ considerably. On the van Houdt dataset, there are 24 proteins with ibb *p*-values *<*0.05 and edgeR *p*-values *>*0.05. Figure 6a lists top five proteins having highest edgeR *p*-values. Here the counts show consistent regulation in all three samples, indicating that the ibb *p*-values are reasonable. Nevertheless, three of five proteins have ibb *p*-value close to the 5% cutoff (*>*0.04), and so are the edgeR *p*-values for most of the 24 proteins in this set.

In the same manner, we examine 17 proteins with edgeR *p*-values *<*0.05 and ibb *p*-values *>*0.05. Figure 6b lists top five proteins having highest ibb *p*-values. Again, the ibb *p*-values in this case are close to the 5% borderline in this set. The second protein in this list does not have consistent regulation, but still has a low edgeR *p*-value. Overall, on the van Houdt dataset, ibb and edgeR have similar performance and both outperform MH for significance analysis (data not shown).

On the Tuch dataset, there are 1664 genes with ibb *p*-values *<*0.05 and edgeR *p*-values *>*0.05, much higher than the number 453 of genes with edgeR *p*-values *<*0.05 and ibb *p*-values *>*0.05. Out of the 1664 genes, we found that ibb can detect significant differences at low fold changes. It is likely due to the random effect model which favors consistent regulation. Figure 6c lists top five proteins (ibb *p*-value *<*0.05 and ibb absolute fold change *>*1.5) having highest edgeR *p*-values. Again, we observe consistent regulation, indicating that the ibb *p*-values are reasonable. The third gene in this list demonstrates observed fold changes of 1.43, 1.44 and 1.57 in the three sample pairs. The fold change returned by edgeR is 1.27, which seems to be an effect of smoothing dispersion across multiple genes.

Figure 6d lists top five proteins (edgeR *p*-value *<*0.05 and ibb absolute fold change *>*1.5) having highest ibb *p*-values. Unlike the result on the van Houdt dataset, here edgeR results appear counterintuitive as all five genes show mixed regulation with high read counts. This behavior is similar to MH, suggesting that it might be a consequence of the fixed-effect model.

## 5 DISCUSSION

This article addresses the problem of significance analysis for paired samples with count data. This type of statistical testing arises, for example, in studies where one is interested in a treatment effect or when one plans to correct for differences in genetic background by using matched cancer/normal tissues.

By formulating the problem in the framework of combining contingency tables, we can use a large body of literature in statistical meta-analysis. For instance, the forest plot, a frequently used visualization method in meta-analysis, offers a dedicated visualization tool for paired sample tests.

We have proposed a novel statistical test using an inverted beta-binomial distribution, explicitly separating technical variation from biological variation. This is in contrast to a state-of-the-art technique, an extension of the edgeR method, which models total variation with a negative binomial distribution. Experimental results on a proteomics dataset and a genomics dataset demonstrate that ibb is a considerable alternative to the extension of edgeR as it tends to favor unidirectional regulation.

Technical variation modeled by the ibb test can be adapted to account for over or under dispersion using an exponentiated Poisson distribution. In theory, one should examine the acquisition platform from independent studies to accurately capture the technical variation. This is, however, not always possible in practice since typically data acquisition comprises of several steps in a complex workflow, in which technology platforms such as a mass spectrometer or a sequencer play a part only. Hence, one needs to make assumptions about the data generative mechanism, for which the standard Poisson distribution provides a reasonable approximation and is a common practice. Deviating from the standard Poisson distribution should be treated with special care.

A generalized linear mixed model (GLMM) is a natural statistical framework to account for random effect (Agresti, 2002). The main difficulty with model fitting in GLMM is that the likelihood function does not have a closed form and thus one has to resort to complicated numerical methods for optimization (Breslow and Clayton, 1993; McCulloch, 1997), similar to the challenge we face with ibb. We have shown that for one-dimensional integration, approximation by a numerical quadrature is efficient and accurate. In addition, when *t_a_* = *t_b_*, a closed form without integration exists for ibb and suited optimization is available.

Bayesian modeling offers another approach to the problem. The modeling can be either for individual proteins/genes or for the complete dataset where the concept of false discovery rate can be incorporated. Once a model has been specified, generic software packages can be used to compute/simulate a posterior distribution given the data and to calculate values such as the Bayes factors to rank proteins/genes. Judging the pros and cons of Bayesian methods goes beyond the scope of this article.

One could also perform the Fisher's exact test for each pair and subsequently combine the resulting *p*-values, for example using the (meta-analysis method in Lu *et al.* (2010)). However, this approach lacks an estimation of the common fold change, which is often used in practice in combination with the *p*-value to select differential candidates.

Finally, software for the inverted beta-binomial test is available as an R package.

*Funding:* VUmc-Cancer Center Amsterdam.

*Conflict of Interest:* none declared.
